# Dietary Intake of Micronutrients and Disease Severity in Patients with Amyotrophic Lateral Sclerosis

**DOI:** 10.3390/metabo13060696

**Published:** 2023-05-27

**Authors:** Acsa Nara de Araújo Brito Barros, Maria Luisa do Nascimento Felipe, Isabelle Ribeiro Barbosa, Lucia Leite-Lais, Lucia Fátima Campos Pedrosa

**Affiliations:** 1 Postgraduate Program in Health Sciences, Health Sciences Center, Federal University of Rio Grande do Norte, Natal 59078-970, RN, Brazil; acsa.barros@ufrn.br; 2 Postgraduate Program in Nutrition, Health Sciences Center, Federal University of Rio Grande do Norte, Natal 59078-970, RN, Brazil; maria.luisa.felipe.111@ufrn.edu.br; 3 Faculty of Health Sciences of Trairi (FACISA), Federal University of Rio Grande do Norte, Santa Cruz 59200-000, RN, Brazil; isabelleribeiro68@gmail.com; 4 Department of Nutrition, Federal University of Rio Grande do Norte, Natal 59078-970, RN, Brazil; lucia.leite@ufrn.br

**Keywords:** amyotrophic lateral sclerosis, micronutrients, food intake

## Abstract

Vitamins and essential metals have been studied as potential risk and prognostic factors in amyotrophic lateral sclerosis (ALS). This study aimed to evaluate the prevalence of inadequate micronutrient intake in ALS patients, comparing subgroups according to the disease severity. Data were obtained from the medical records of 69 individuals. Assessment of disease severity was determined by the revised ALS Functional Scale (ALSFRS-R), using the median as the cutoff. The prevalence of inadequate micronutrient intake was estimated using the Estimated Average Requirements (EAR) cut-point method. The prevalence of inadequate vitamin D, E, riboflavin, pyridoxine, folate, cobalamin, calcium, zinc, and magnesium intake was considered severe. Patients with lower ALSFRS-R scores had lower intakes of vitamin E (*p* < 0.001), niacin (*p* = 0.033), pantothenic acid (*p* = 0.037), pyridoxin (*p* = 0.008), folate (*p* = 0.009) and selenium (*p* = 0.001). Therefore, ALS patients should be monitored regarding dietary intake of micronutrients essential in neurological processes.

## 1. Introduction

Amyotrophic lateral sclerosis (ALS) is a fatal neurodegenerative disease that affects upper and lower motor neurons, producing a progressive weakness of skeletal muscles involved in limb movement, swallowing, speech and respiratory function [1].

The major risk factors for the development of ALS are genetics, prolonged exposure to toxic metals and pollutants, and lifestyle [2]. Nutritional status has been studied as risk and prognostic factors in ALS. Body composition [3,4], malnutrition at diagnosis [5,6], acquired malnutrition in the course of the disease [7], and macronutrients intake [8] seem to influence the survival of patients with ALS. Also, vitamins [9,10,11] and essential metals [12,13,14] have been studied as potential risk and prognostic factors in ALS.

In a Finnish cohort, higher baseline levels of vitamin E were associated with a lower subsequent risk of ALS [15]. This protective effect of vitamin E against ALS appears to be related to antioxidant protection against increased levels of free radicals and lipid peroxidation, scaling down neuroinflammation [9,11]. Recently, a Mendelian randomization analysis found that increased blood levels of vitamin E and D appear to be protective against ALS risk [11]. In ALS, vitamin D potentiates the effect of neurotrophic factors and protects motor neurons. In addition, vitamin D upregulates calcium-binding proteins and glutamate-induced reduction of caspase-3 activity, leading to neuroprotection [9,11]. Based on results from meta-analysis, randomized clinical trials, and clinical cases, vitamins B12 and C have also been shown to be protective against ALS risk [9]. However, despite the results showing suggestive conclusions about the protective role of such vitamins against the risk of ALS, they are still inconclusive [16,17,18,19].

The role of metals in ALS varies according to their beneficial or toxic effects. Elevated serum levels of zinc [20], copper, and iron [14] are suggested as potential risk factors for ALS. Furthermore, a recent meta-analysis found that mean serum levels of selenium were significantly higher in ALS patients compared to controls [21]. These metals are known cofactors for enzymes necessary for proper functioning of the central nervous system, but in excess, they can be toxic [14,20,21]. Despite these findings, associations between the concentration of some metals in body fluids and ALS etiology are still inconclusive [22].

The ALS prognosis also seems to be related to some micronutrients. Vitamin D deficiency was associated with a faster progression rate (ΔFS) of disease in ALS patients [23,24]. In neuronal cell culture, vitamin D promotes motoneuron survival by potentiating the activity of neurotrophic factors and blocking death receptors [23]. In addition, intramuscular high-dose methylcobalamin decreased the progression of ALS, measured by the Revised Amyotrophic Lateral Sclerosis Functional Rating Scale (ALSFRS-R) [25]. A possible mechanism for this may be related to the effects caused by vitamin B12 deficiency, which can lead to hyperhomocysteinemia. In excess, homocysteine has a neurotoxic effect by increasing oxidative stress and contributing to neuronal degeneration in patients with ALS. Vitamin B12 also plays vital roles in deoxyribonucleic acid synthesis, epigenetic modification, methylation, and mitochondrial function [25,26]. In contrast, high calcium and copper levels in the blood were related to a high ALSFRS-R score [14]. In fact, copper and calcium imbalance has been implicated in various neurodegenerative diseases. In ALS, increased calcium is related to oxidative stress, mitochondrial dysfunction, excitotoxicity, and neuroinflammation [27].

A cross-sectional baseline analysis of an American cohort showed that a high intake of antioxidants and carotenoids from vegetables was associated with a higher ALSFRS-R score [28]. A similar result was observed in a cross-sectional study with ALS patients in South Korea in which the intake of vitamin D, vitamin E, thiamine, riboflavin, pyridoxine, niacin, folate, calcium, phosphorus, sodium, potassium, iron, zinc, copper, and manganese was significantly lower in patients with a lower ALSFRS-R score, although significance disappeared after adjustment for energy [29].

Although micronutrients have been studied in relation to ALS risk and prognosis, the results are still inconsistent indicating a need for more studies in this matter. Therefore, the aim of this study was to evaluate the prevalence of inadequate micronutrient intake in ALS patients, comparing subgroups according to the disease severity.

## 2. Materials and Methods

### 2.1. Study Design and Population

This cross-sectional study was conducted at the Multidisciplinary ALS Outpatient Clinic of the Onofre Lopes University Hospital (HUOL), Natal/RN, Brazil. The project was approved by the Ethics Committee of HUOL-UFRN (CAEE 21921219.1.0000.5292).

We included adult and elderly patients of both genders, with probable or definite ALS diagnosis [1] and whose food record data were present in their medical records. We excluded individuals with a suspected or possible ALS diagnosis, patients with alternative feeding route (gastrostomy), or who had another neurological disease, food allergies and intolerances, inflammatory bowel diseases, diabetes mellitus, and kidney diseases, due to the possible interference of these variables in food intake by restricting specific foods. Data collection was performed between December 2019 and January 2020. Information from 69 patients with ALS regarding the clinical, anthropometric, and dietary characteristics were obtained from medical records.

### 2.2. Clinical Assessment

The ALS severity was determined by the ALSFRS-R [30], which determines the degree of impairment in ALS patients’ abilities to function independently in activities of daily living. The ALSFRS-R measures 12 aspects of physical function and each function is scored from 0 to 4, with a maximum total score of 48 (normal) and a minimum total score of 0 (no ability). Patients were assessed according to all 12 aspects of the scale and the total ALSFR score was obtained. The ALSFRS-R score was also used to calculate the ΔFS, from the equation [31,32]:

$$\Delta \mathrm{FS}=\frac{48-(Total\;ALSFRS-R\;score\;at\;initial\;assessment)}{Time\;from\;onset\;symptoms\;to\;date\;of\;initial\;assessment\;in\;months}$$

The medians of the ALSFRS-R and ΔFS were used as the cutoff point to classify the sample into lower (below median) and higher (equal to or above median) scores, and slow (below median) and rapid (equal to or above median) progression, respectively. Dichotomization based on our median data was performed, as the value may change among studies [31,32,33].

### 2.3. Dietary Assessment

The usual dietary energy, macronutrients and micronutrient intake was determined according to the mean values of intake registered in the two non-consecutive 24-h dietary recalls (R24 h). Data were analyzed using the Virtual Nutri Plus^®^ 2.0 software (São Paulo, SP, Brazil) software. Nonexistent foods were added to the software’s database as necessary, based on the nutrition labels. Then, within-person variability of dietary intake was removed using the Multiple Source Method (MSM) [34] and the adjustment for energy was performed by the residue method [35].

Mean energy and macronutrient intake were compared to specific ALS recommendations used in our outpatient clinic [36]. The prevalence of inadequate micronutrient intake was estimated according to sex and age using the Estimated Average Requirements (EAR) cut-point method [37,38]. The prevalence of inadequate iron intake was performed by a probabilistic approach [37,38]. Pantothenic acid, manganese, potassium, and sodium were assessed based on Adequate Intake (AI). The prevalence of inadequate micronutrient intake was classified as no problem (≤4.9%), mild (5.0–19.9%), moderate (20.0–39.9%), and severe (≥40.0%) [39].

### 2.4. Statistical Analysis

Skewness and Kurtosis tests were used to assess the data normality. Differences in clinical characteristics and micronutrient intake between the groups formed from the median ALSFRS-R score were calculated using the t-student, Mann-Whitney U, Chi-square, and Fisher’s Exact tests, according to the nature and distribution of the variable. Differences with *p* < 0.05 were considered statistically significant. All analyses were performed using SPSS 25.0 software (Chicago, IL, USA).

## 3. Results

Study participants had a median age of 56.0 (13.6) years, BMI of 23.7 (3.3) kg/m^2^, and median duration of symptoms of 25.5 (1.3–248) months. Most participants were male (61%) and had spinal onset of the disease (75%). The ΔFS indicated that 50% of the sample had rapid progression and the other half had slow progression. When compared to higher or lower ALSFRS-R scores, most patients with slow ΔFS had higher scores on the scale (62%), although the difference was not statistically significant (Table 1).

**Figure 1:**
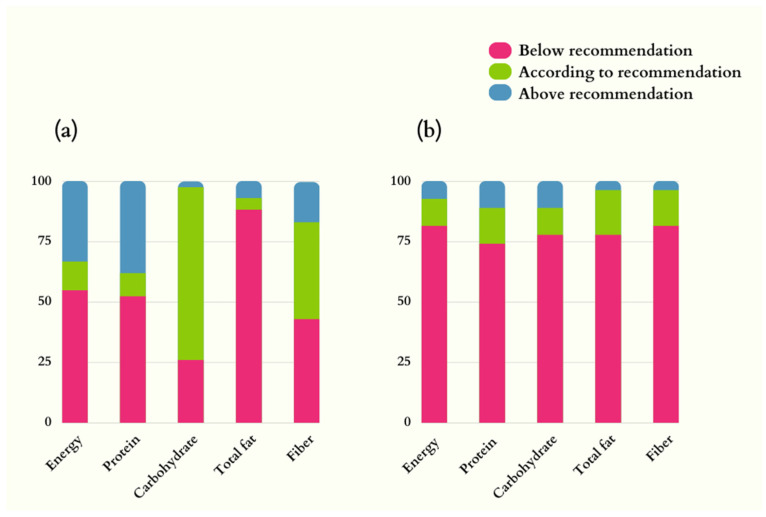
Dietary intake of energy, macronutrients, and fiber in ALS patients. (**a**) Men (n = 42); (**b**) Women (n = 27). Nutrition recommendations adopted: energy = 35 kcal/kg/d; protein = 1.5 g/d; lipids = 30% of the total energy value; carbohydrates = remaining percentage to complete the total energy value; fibers: 20–30 g/d [36].

Dietary energy intake was below recommended levels in 54.8% of men and 81.5% of women. Below-recommended intake in both sexes was also observed for protein (52.4% men and 74.1% women) and total fat (88.1% men and 77.8% women). Most of the male participants (71.4%) ingested carbohydrates according to the recommendation levels for ALS patients (Figure 1).

The prevalence of inadequate intake of micronutrients in women was considered severe for vitamin D (99%), vitamin E (40%), riboflavin (55%), pyridoxine (42%, >50y), folate (77%), cobalamin (40%), calcium (68%, 19–50y; 78%, >50y), zinc (46%), and magnesium (64%) (Table 2). In men, severe prevalence of inadequate intake was observed for vitamin D (99%), riboflavin (62%), pyridoxine (52%, >50y), folate (82%), cobalamin (41%), calcium (61%, 19–70y), zinc (49%) and magnesium (98%) (Table 3).

Significant differences were observed in micronutrient intake between the groups according to the ALSFRS-R score. Patients with higher scores had higher intakes of vitamin E (*p* < 0.001), niacin (*p* = 0.033), pantothenic acid (*p* = 0.037), pyridoxine (*p* = 0.008), folate (*p* = 0.009) and selenium (*p* = 0.001), compared to patients with lower ALSFRS-R scores (Table 4).

## 4. Discussion

In our study, some vitamins and essential metals with an important role in ALS showed a severe prevalence of inadequate intake. For example, intake of vitamin E, niacin, pantothenic acid, pyridoxine, folate, and selenium was lower in the group with the lowest ALSFRS-R score. Whereas the functional impairment of the patient with ALS can interfere with the adequate intake of nutrients, micronutrient deficiency, in turn, can play a negative role in the neurodegenerative processes [40]. 

The severe prevalence of inadequate vitamin D intake in our study, added to the difficulty of exposure to the sun in more advanced stages of ALS, is concerning. Deficient levels of vitamin D (<20 ng/mL) have been found in patients with ALS [19,41,42]. In animal models of ALS, low vitamin D intake exacerbated the disease’s pathophysiology by increasing inflammation and oxidative damage and reducing antioxidant capacity [43]. Although associations between vitamin D and ALS prognosis remain inconclusive [19,23,24], the organic functions performed by the vitamin in the neurological context must be considered [9,40,44], as well as adequate supplementation [41] when food intake and sun exposure are inevitably inefficient to meet the needs of patients with ALS. 

In this study, the prevalence of inadequate vitamin E intake was also considered severe. Furthermore, the lowest intake occurred among patients with the lowest ALSFRS-R score. Although the associations between vitamin E and the ALS prognosis remain controversial, the antioxidant property of this vitamin may contribute to the reduction of neuronal damage and delay the neurodegenerative process of ALS [18,45]. Even with no solid rationale for indiscriminate vitamin E supplementation to slow disease progression, maintaining adequate vitamin E intake in these patients should be prioritized, especially in patients with reduced ALSFRS-R scores.

B Vitamins are essential in macronutrient metabolism, gene regulation, neurotransmitter synthesis, and antioxidants [46]. Our study found a severe prevalence of inadequate intake of thiamine, riboflavin, folate, pyridoxine, and cobalamin. Additionally, we observed that the intake of pyridoxine, folate, niacin, and pantothenic acid was lower in patients with lower ALSFRS-R scores. A deficiency of one or more B vitamins leads to neurological impairment [9,46,47,48]. In patients with ALS, elevated homocysteine levels in the plasma and cerebrospinal fluid have been found. Elevated homocysteine has neuropathologic activity and can be reversed by an adequate supply of folate and cobalamin [26,49]. Therefore, adequate nutrient intake that supports an already weakened neurological system must be addressed. It is also essential to consider the hydro-solubility of these vitamins, which may incur bioavailability losses in food processing [50]. Thus, in patients with compromised functional capacity, attention should be paid to niacin, pantothenic acid, pyridoxine, and folate intake.

Zinc is an essential metal in the structure and activity of several enzymes. Studies evaluating the relationship between zinc and the risk and prognosis of ALS are contradictory [51]. However, a recent systematic review concluded that environmental exposure to zinc is a factor strongly associated with ALS risk [52]. Accordingly, a prospective observational study found an association between an increased risk of ALS with increased zinc intake [53]. In our study, patients with a higher ΔFS had a higher zinc intake (Table S1). 

In contrast, higher RBC zinc levels in a European cohort were associated with a decreased risk of ALS [20]. Higher zinc levels in whole blood were also inversely associated with ALS, especially among patients with poor functional capacity [13]. A Brazilian cross-sectional study found zinc deficiency in ALS patients [54]. Contradictorily, in a sample of patients with sporadic ALS in China, zinc blood levels did not differ from the control group [14]. It is essential to highlight that one of the factors involved in the etiology of hereditary ALS, which comprises 20% of ALS cases, is the protein aggregates formed due to the genetic mutation of superoxide dismutase 1 (SOD1), an enzyme dependent on copper and zinc [1]. Zinc loss in SOD1 induces neuronal death and may have a causal role in familial ALS [55]. Although studies are controversial, zinc is related to ALS and adequate intake is advised.

Another essential metal of interest in ALS is selenium due to its antioxidant properties and role in hormonal functions, immunity, and inflammatory response [51]. While lower levels of selenium in whole blood were inversely associated with ALS risk [13], environmental exposure to the metal may have a causal effect on the disease [22]. However, some authors hypothesize that selenium plays a protective role in ALS, due to its role as an antioxidant in the central nervous system and the fact its deficiency causes neurological damage [51]. Recognition of the benefits of selenium in ALS, however, is not hegemonic. Even in the face of the biological plausibility of their functions, the contradictory results of studies do not ensure a solid conclusion [56,57,58,59].

The prevalence of inadequate selenium intake was considered moderate in our sample, and patients with worse function ingested less selenium. Although there is no confirmation of the role of selenium in the progression of ALS, dietary intake of this metal is required for maintaining adequate brain levels of the antioxidant enzyme selenoprotein P [60,61,62]. Vigilance regarding the intake of this essential metal for proper neurological functioning is required in patients with lower ALSFRS-R scores.

Other essential metals such as magnesium and calcium had a prevalence of severe inadequate intake. Data on the role of magnesium in the risk and progression of ALS remain inconclusive, despite its essential role in neural and neuromuscular transmission [22,51,63]. Elevated serum calcium levels in ALS patients have been reported in the literature [51] and positively correlated with high ALS severity, although the results are still inconclusive [14]. Furthermore, dysregulation of calcium homeostasis related to mitochondrial dysfunction is a pathogenetic mechanism of ALS [64,65]. However, dietary intake of these elements does not appear to be related to ALS progression and risk [26,53,63].

In general, studies involving antioxidant micronutrients in ALS show a possible association with lower risk and better motor function [66]. However, consuming food sources of micronutrients does not necessarily imply their absorption and use by the body. Ingested amounts, chemical form, interactions with other nutrients, in addition to homeostatic mechanisms that regulate absorption, all can interfere with nutrient bioavailability [67]. Consequently, promoting adequate nutritional intake in these patients is fundamental.

The prevalence of severe intake of nutrients observed in our sample considered the EAR cut-point method. The EAR was conceived as a value that meets the daily requirement of a nutrient in only 50% of healthy individuals [37]. Thus, it is possible that the real needs of our patients have been underestimated. Due to the lack of specific recommendations for micronutrient intake to ALS patients, in individualized care we recommend the use of the Recommended Dietary Allowance (RDA), whose values were designed to meet the needs of 97% to 98% of healthy individuals [36,37].

Nutritional needs differ between the sexes due to large differences in their physiological and hormonal states [68,69,70]. Added to this, sex influences the phenotypic presentation of ALS. Women aged 60 years and older commonly develop bulbar onset [1], which directly affects nutrient intake early in the disease. This result adds one more variable to the impairment of nutritional status, as patients with ALS have unintentional weight loss caused by muscle atrophy and hypermetabolism, capable of leading these patients to a state of malnutrition [71]. In addition, impairment of muscles involved in mastication and swallowing, modification in diet consistency, changes in appetite, lack of adequate assistance and psychological factors also affect the food intake of ALS patients [71,72]. 

The damage caused by energy intake below the recommendations goes beyond the commitment of the anthropometric nutritional status, as it directly affects the intake of micronutrients, since the consumption of most nutrients is associated with the total energy intake [35]. Thus, dietary counseling for patients with ALS should include adequate caloric intake, micronutrient intake, dietary changes and, in some cases, the use of gastrostomy, since the nutritional status of patients with ALS directly interferes with survival [66,73].

A limitation in our study was the lack of biomarkers for vitamins and essential metals in ALS patients, indicators of nutritional status that could help in understanding the association between low micronutrient intake and prognosis, measured by ALSFRS-R and ΔFS. A strong point of our study was the use of appropriate statistical methods to obtain a more accurate estimate of usual nutrient intake and the prevalence of inadequate micronutrient intake.

## 5. Conclusions

In our study, the prevalence of inadequate intake of micronutrients was considered severe for vitamin D, vitamin E, riboflavin, pyridoxine, folate, cobalamin, calcium, zinc, and magnesium. Also, the disease severity of patients with ALS assessed by the ALSFRS-R score was related to the lower intake of vitamin E, niacin, pantothenic acid, pyridoxine, folate and selenium, which are important micronutrients in the context of ALS. Given the factors that interfere with food intake, patients with ALS are more likely to have inadequate dietary intake of micronutrients. In view of this, periodic nutritional assessment should include monitoring micronutrient intake, either preventively or correctively.

## Figures and Tables

**Table 1:** Clinical characteristics of participants according to ALSFRS-R score.

Variables	Total (n = 69)	ALSFRS-R Score		*p*-Value
		≥34 (n = 36)	< 34 (n = 33)	
Age, years ^a^	56.0 (13.6)	54.5 (14.4)	57.3 (12.7)	0.362 *
BMI, kg/m^2 a^	23.7 (3.3)	23.9 (3.5)	23.4 (3.2)	0.546 *
Age at onset (years) ^a^	53.0 (14.4)	52.0 (15.8)	53.8 (13.0)	0.576 *
Symptom duration (months) ^b^	25.5 (1.3-248)	31.1 (48.2)	45.5 (35.7)	0.166 **
Gender ^c^				
Female	27 (39)	10 (37)	17 (63)	0.052 †
Male	42 (61)	26 (62)	16 (38)	
Site of onset ^c^				
Bulbar	17 (25)	10 (59)	7 (41)	0.585 †
Spinal	52 (75)	26 (50)	26 (50)	
ΔFS ^cd^				
Slower (<0.66)	34 (50)	21 (62)	13 (38)	0.145 †
Faster (≥0.66)	34 (50)	14 (41)	20 (59)	
ALS family history ^c^				
No	63 (91)	34 (54)	29 (46)	0.416 ††
Yes	6 (9)	2 (33)	4 (67)	
Use of medication (Riluzole) ^c^				
No	65 (94)	34 (52)	31 (48)	1.00 ††
Yes	4 (6)	2 (50)	2 (50)	

^a^ Mean (standard deviation); ^b^ median (interquartile range); ^c^ frequency (%); ^d^ median used as cutoff; * Student’s *t*-test for independent samples; ** Mann-Whitney U-test; † Square test; †† Fisher’s exact test; ΔFS, progression rate.

**Table 2:** Nutritional recommendation, dietary intake, and prevalence of inadequate micronutrient intake in women with amyotrophic lateral sclerosis.

Micronutrients	EAR/AI *	Mean (SD)	Intake Percentiles					% Of Inadequacy
			10th	25th	50th	75th	90th	
Vitamin A, μg/d	500	1376 (2537)	193	470	715	1310	2069	36
Vitamin C, mg/d	60	200 (107)	95	124	162	238	360	10
Vitamin D, μg/d	10	4.3 (2.4)	0.9	3.3	4.2	5.3	7.5	99
Vitamin E, mg/d	12	15 (8.9)	2.6	8.1	15	23	29	40
Thiamin, mg/d	0.9	2.0 (0.4)	1.5	1.7	1.8	2.2	2.4	0.5
Riboflavin, mg/d	0.9	0.8 (0.9)	0.2	0.3	0.5	1.1	1.6	55
Niacin, mg/d	11	18 (9.2)	8.6	12	15	23	32	22
Pantothenic Acid, mg	5 *	3.4 (2.4)	1.0	1.4	3.1	4.6	6.2	-
Pyridoxin, mg/d								
19–50y	1.1	1.9 (0.7)	0.7	1.5	1.8	2.7	-	13
>50y	1.3	1.4 (0.7)	0.5	0.9	1.4	2.1	2.3	42
Folate, mcg/d	320	214 (144)	48	105	189	297	469	77
Cobalamin, mcg/d	2	9.9 (31)	0.7	2.1	3.3	4.6	8.0	40
Phosphorus, mg/d	580	1055 (182)	812	953	1010	1156	1363	0.5
Calcium, mg/d 19–50y >50y	800 1000	649 (266) 780 (290)	324 405	487 575	600 750	794 898	- 1208	68 78
Iron, mg/d 19–50y >50y	8.1 5	14 (4.0) 16 (6.1)	8.1 10	11 11	14 14	15 20	- 24	14 2.3
Zinc, mg/d	6.8	9.9 (3.1)	7.2	7.8	9.0	11	14	46
Copper, mcg/d	0.7	1.5 (1.0)	0.8	0.9	1.3	1.7	2.5	22
Potassium, mg/d	2600 *	2240 (466)	1516	1982	2225	2541	2856	-
Magnesium, mg/d	265	241 (62)	181	206	232	255	337	64
Selenium, μg/d	45	81 (105)	17	28	65	90	156	37
Manganese, mg/d	1.8 *	1.9 (0.7)	1.1	1.5	1.8	2.0	3.0	-

*AI, Adequate Intake; EAR, Estimated Average Requirement.

**Table 3:** Nutritional recommendation, dietary intake, and prevalence of inadequate micronutrient intake in men with amyotrophic lateral sclerosis.

Micronutrients	EAR/AI *	Mean (SD)	Intake Percentiles					% of Inadequacy
			10th	25th	50th	75th	90th	
Vitamin A, μg/d	625	1445 (2414)	431	577	869	1212	2704	37
Vitamin C, mg/d	75	277.1 (405.0)	130	139	169	263	497	31
Vitamin D, μg/d	10	4.8 (2.2)	2.0	3.2	4.9	6.5	7.3	99
Vitamin E, mg/d	12	15.4 (8.1)	2.5	11	16	21	26	34
Thiamin, mg/d	1	1.9 (0.4)	1.4	1.7	1.9	2.1	2.6	0.8
Riboflavin, mg/d	1.1	0.8 (0.9)	0.2	0.3	0.7	1.1	1.5	62
Niacin, mg/d	12	17 (7.5)	8.6	12	15	22	29	25
Pantothenic Acid, mg	5 *	3.1 (1.8)	1.2	2.0	3.2	3.8	5.0	-
Pyridoxin, mg/d 19–50y >50y	1.1 1.4	1.7 (0.8) 1.4 (0.5)	0.5 0.7	0.9 1.0	1.4 1.3	2.1 1.6	2.3 2.4	25 52
Folate, mcg/d	320	198 (140)	32	119	193	262	371	82
Cobalamin, mcg/d	2	9.1 (31)	1.6	2.4	3.7	4.9	6.5	41
Phosphorus, mg/d	580	1080 (205)	800	973	1070	1165	1350	0.8
Calcium, mg/d 19–70y >70y	800 1000	707 (304) 1177 (266)	364 972	492 988	635 1086	855 1457	1151 -	61 25
Iron, mg/d	6	13 (8.4)	7.2	10	12	16	20	4.9
Zinc, mg/d	9.4	9.6 (2.9)	6.2	7.7	9.3	11	13	49
Copper, mcg/d	0.7	1.5 (1.1)	0.7	1.0	1.3	1.7	2.5	23
Potassium, mg/d	3400 *	2303 (521)	1669	2045	2243	2610	2991	-
Magnesium, mg/d	350	229 (61)	144	198	216	261	308	98
Selenium, μg/d	45	53 (32)	9.3	30	52	71	96	39
Manganese, mg/d	2.3 *	1.7 (0.7)	0.9	1.1	1.6	2.2	2.5	-

*AI, Adequate Intake; EAR, Estimated Average Requirement.

**Table 4:** Differences in usual dietary intake of micronutrients according to ALSFRS-R score.

Variable	ALSFRS-R Score ^a^		*p*-Value
	≥34 (n = 36)	<34 (n = 33)	
Vitamin A, μg/d	1020 (3386)	715 (687)	0.361
Vitamin C, mg/d	152 (451)	197 (100)	0.140
Vitamin D, μg/d	4.5 (2.2)	4.7 (2.4)	0.505
Vitamin E, mg/d	19 (7.9)	10 (6.9)	<0.001
Thiamin, mg/d	1.9 (0.3)	1.8 (0.4)	0.065
Riboflavin, mg/d	0.7 (1.1)	0.6 (0.5)	0.255
Niacin, mg/d	18 (8.3)	13 (7.8)	0.033
Pantothenic Acid, mg	3.4 (2.0)	2.2 (2.1)	0.037
Pyridoxin, mg/d	1.8 (0.7)	1.2 (0.6)	0.008
Folate, mcg/d	223 (146)	139 (125)	0.009
Cobalamin, mcg/d	4.1 (43)	3.3 (4.0)	0.121
Phosphorus, mg/d	1067 (165)	1061 (225)	0.895
Calcium, mg/d	627 (312)	822 (307)	0.079
Iron, mg/d	13 (9.2)	12 (4.7)	0.073
Zinc, mg/d	9.1 (2.4)	9.2 (3.1)	0.266
Copper, mcg/d	1.3 (1.3)	1.2 (0.8)	0.749
Potassium, mg/d	2144 (463)	2277 (552)	0.349
Magnesium, mg/d	208 (51)	230 (73)	0.355
Selenium, μg/d	64 (91)	35 (35)	0.001
Manganese, mg/d	1.6 (0.5)	1.8 (0.8)	0.057

Data presented as mean (standard deviation); ^a^ median as cutoff; Independent sample U Mann-Whitney test.

## Data Availability

The datasets used and/or analysed during the current study are available from the corresponding author on reasonable request due to privacy.

## Supplementary Materials

The following supporting information can be downloaded at: https://www.mdpi.com/article/10.3390/metabo13060696/s1, Table S1: Differences in usual intake of micronutrients according to progression rate.
