# Supplementary material for: Dietary Intake of Micronutrients and Disease Severity in Patients with Amyotrophic Lateral Sclerosis
Source: Metabolites. 2023 May 27;13(6):696. doi: 10.3390/metabo13060696 (PMC10302905; doi:10.3390/metabo13060696)
Supplement: Supplementary file 1 [file metabolites-13-00696-s001.zip › metabolites-2400440-supplementary.pdf]

Dietary intake of micronutrients and disease severity in patients with amyotrophic lateral sclerosis

Acsa Nara de Araújo Brito Barros, Maria Luisa do Nascimento Felipe, Isabelle Ribeiro Barbosa, Lucia Leite-Lais and Lúcia Fátima Campos Pedrosa

Supplementary Material

Table S1. Differences in usual dietary intake of micronutrients according to ALS progression rate.

Variable	ΔFS[a] Faster ≥ 0.66 (n=34)	ΔFS[a] Slower < 0.66 (n=34)	*p*-value *
Vitamin A, µg/d	778 (2733)	1069 (2358)	0.315
Vitamin C, mg/d	163 (457)	160 (125)	0.713
Vitamin D, µg/d	4.7 (2.2)	4.3 (2.4)	0.792
Vitamin E, mg/d	15 (8.0)	15 (8.7)	0.898
Thiamin, mg/d	1.8 (0.4)	1.9 (0.4)	0.995
Riboflavin, mg/d	0.6 (0.9)	0.7 (0.9)	0.291
Niacin, mg/d	17 (9.9)	15 (6.1)	0.477
Pantothenic Acid, mg	3.0 (2.0)	3.4 (2.2)	0.699
Pyridoxin, mg/d	1.6 (0.7)	1.4 (0.6)	0.649
Folate, mcg/d	164 (131)	185 (137)	0.966
Cobalamin, mcg/d	3.30 (35)	3.7 (28.6)	0.316
Phosphorus, mg/d	1029 (177)	1069 (200)	0.342
Calcium, mg/d	635 (289)	797 (325)	0.073
Iron, mg/d	13 (5.0)	12 (9.7)	0.297
Zinc, mg/d	8.3 (2.4)	10 (2.9)	0.005
Copper, mcg/d	1.3 (1.1)	1.3 (1.1)	0.936
Potassium, mg/d	2227 (495)	2248 (491)	0.741
Magnesium, mg/d	211 (61)	235 (61)	0.659
Selenium, µg/d	55 (96)	43 (39)	0.778
Manganese, mg/d	1.7 (0.5)	1.6 (0.8)	0.468

Data presented as mean (standard deviation); [a]median as cutoff; *The significance level is 0.05; Independent sample U Mann-Whitney test; ΔFS, progression rate
